# Teledermatology in the Control of Skin Neglected Tropical Diseases: A Systematic Review

**DOI:** 10.5826/dpc.1104a130

**Published:** 2021-10-01

**Authors:** Tejas P. Joshi, Vicky Ren

**Affiliations:** 1 School of Medicine, Baylor College of Medicine; 2 Department of Dermatology, Baylor College of Medicine

**Keywords:** global dermatology, global health, skin neglected tropical diseases, teledermatology, telemedicine

## Abstract

**Introduction:**

Neglected tropical diseases (NTDs) include a group of about 20 illnesses that have garnered relatively little attention, despite their ability to inflict significant suffering and disability. Skin neglected tropical diseases (sNTDs) are a subset of NTDs that present with cutaneous manifestations and are well known for their ability to generate stigma and promote poverty. Teledermatology (TD) represents a potential method to control sNTDs.

**Objective:**

We sought to analyze the potential for TD to ease the burden of sNTDs.

**Methods:**

We performed a systematic literature search using the Texas Medical Center Library One Search, which scans 167 databases, including Embase, PubMed, and Scopus. We included all original investigations published after 2011 that assessed the impact of TD intervention in the control of one or more sNTDs. We excluded studies not written in English and studies that did not perform any outcome analyses.

**Results:**

Twenty studies met our search criteria, and 18 expressed positive attitudes towards TD. Overall, we found that TD may be a sustainable, cost-effective strategy for expanding access to care for individuals afflicted with sNTDs. However, poor image quality, lack of access to further diagnostic tests, and ethical, legal, and cultural issues pose as barriers to TD utilization.

**Conclusion:**

TD may be helpful in achieving control of sNTDs but has its limitations. An integrated approach, which employs TD in conjunction with other strategies, represents a realistic path for alleviating sNTDs.

## Introduction

Neglected tropical diseases (NTDs) include about 20 debilitating illnesses that affect the world’s most indigent, engendering disability, and suffering [1]. As opposed to HIV/AIDS, tuberculosis, and malaria, NTDs have garnered relatively little attention and funding [2]. Nonetheless, the burden of NTDs is significant, with approximately 1 billion people afflicted, resulting in a loss of roughly 26 million disability-adjusted life-years [3]. Skin neglected tropical diseases (sNTDs) constitute a subset of NTDs that present with cutaneous manifestations. As identified by the World Health Organization (WHO), they include Buruli ulcer (BU), cutaneous leishmaniasis (CL), fungal diseases, leprosy, lymphatic filariasis (LF), mycetoma, onchocerciasis, post-kala-azar dermal leishmaniasis, scabies, and yaws [4]. sNTDs are particularly stigmatizing and lead to marginalization of afflicted individuals, trapping them in a vicious cycle of poverty and disease progression [5].

Teledermatology (TD) may be a powerful tool in facilitating the eradication of sNTDs. In the highly visual field of dermatology, TD has been touted as a cost-effective, time-efficient option for care delivery. Moreover, TD offers 2 flexible models of care: synchronously, over a videoconferencing platform and asynchronously, through photographs sent via online communication tools (store and forward dermatology) [6]. Both synchronous and asynchronous TD models have been extensively deployed during the coronavirus disease 2019 (COVID-19) pandemic, and despite their deficiencies, represent promising paradigms of delivering remote care [7]. As the post-pandemic utilization of TD will likely grow, it is important to consider how TD can aid in the management of sNTDs; simultaneously, it is worth keeping in mind the limitations of TD.

## Objectives

Considering the potential for TD in the management of sNTDs, we reviewed the literature for cases in which TD was used to manage sNTDs. When possible, we extracted information regarding patient and physician satisfaction with TD, time to receiving a TD diagnosis, concordance between TD and face-to-face (FTF) diagnoses, clinical outcomes, cost of TD consultation, encryption of TD platform used, and adequacy of images submitted.

## Methods

We performed a literature search using the Texas Medical Center Library One Search, which scans 167 databases, including Embase, PubMed, and Scopus. We performed our search on February 27, 2021, using the search criteria “teledermatology” AND “skin neglected tropical disease.” For thoroughness, we performed cross validation with every sNTD listed by the WHO, inputting the search criteria “teledermatology” AND “[sNTD recognized by the WHO].” We limited our search to articles published in 2011 or later. Eligible studies were original investigations in which a TD intervention was implemented to diagnose and/or manage one or more sNTDs. We excluded commentaries, editorials, and reviews that did not present any original data. We also excluded case reports (although we discuss some anecdotally to illustrate proof of concept). Articles not written in English and articles that presented TD along with other interventions (such that the impact of TD alone could not be isolated) were also excluded from analysis. Lastly, we excluded articles that implemented TD in the diagnosis of sNTDs but did not perform any further analyses (eg, concordance measures, clinical improvement, time to diagnosis, etc.).

**Figure 1:**
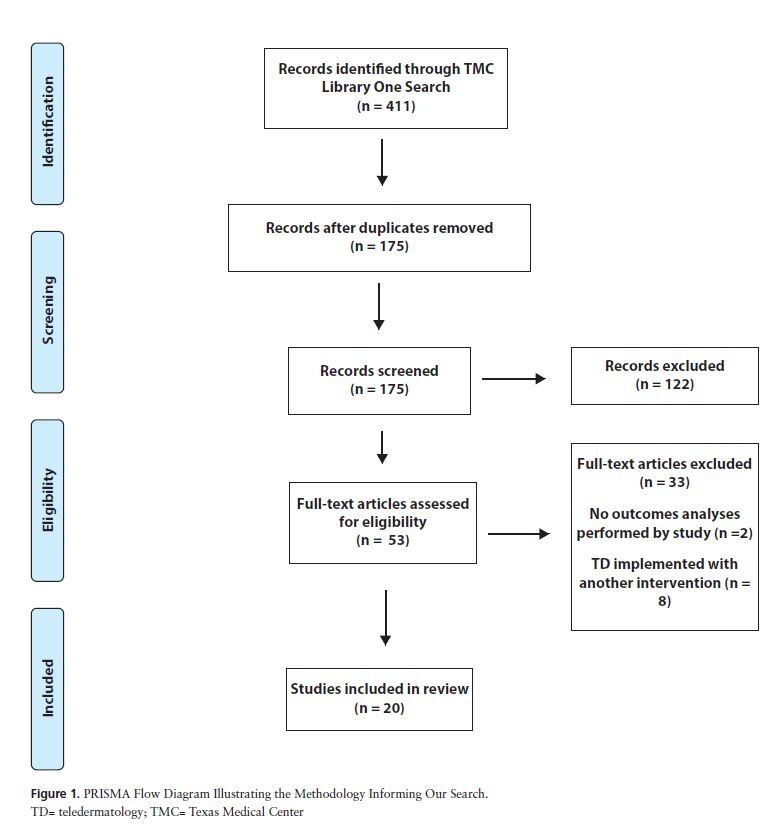
PRISMA Flow Diagram Illustrating the Methodology Informing Our Search. TD= teledermatology; TMC= Texas Medical Center

In preparing this systematic review, we adhered to the Preferred Reporting Items for Systematic Reviews and Meta-Analyses (PRISMA) guidelines. We include a PRISMA flow diagram that illustrates our search (Figure 1).

## Results

Twenty studies met our search criteria (Table 1). Fungal infections were the most common sNTDs to be targeted by TD, being described in 18/20 studies analyzed. Scabies (10/20), CL (5/20), and leprosy (5/20) were the next most common sNTDs to be diagnosed by TD. We did not find any eligible studie that utilized TD in diagnosing mycetoma, onchocerciasis, post-kala-azar dermal leishmaniasis, or yaws. The studies we reviewed had a wide geographical distribution: Latin America (6/20), Africa (7/20), the Middle East (4/20), and Asia (3/20). Interestingly, we also found one study by Hwang et al evaluating TD use in the United States military in deployed settings: while the majority of TD consults were requested from Iraq and Afghanistan, almost 50 locations utilizing TD appointments were described [8]. The majority of the studies we reviewed (18/20) adopted a generally favorable outlook towards TD. Additionally, 5 studies assessed the concordance between FTF and TD consultations, and the agreement ranged from 56% [9] to 95% [10]; the study reporting 56% agreement did not consider this degree of concordance to be sufficient to recommend the independent use of TD [9].

We recognize selection bias as a potential weakness of all the studies we reviewed, as patients uncomfortable with TD would not have elected to participate. Only 3/20 studies documented the number of individuals who declined to participate.

### Teledermatology: Advantages and Promises

Expansion of dermatologic care to underserved regions plagued by sNTDs represents perhaps the most significant benefit of TD. While there is no study that assesses the number of dermatologists per capita by country, data from the United States alone is concerning for sharp disparities between dermatologic care in rural and urban centers, with the difference in dermatologist density between metropolitan and rural counties exceeding 4 dermatologists per 100,000 people [28]. It is likely that such disparities are more pronounced in developing regions where sNTDs constitute a major burden of disease. The studies we reviewed indicate the potential for TD to bridge this gap in access to care, as 12/20 were conducted in rural locations and all were conducted in resource limited settings. Case reports from Nepal support the potential for TD to penetrate rural areas and address sNTDs such as CL [29] and tinea incognito [30]. Formation of a global network of teledermatologists may facilitate the eradication of sNTDs in regions where in-person care may not be feasible.

Additionally, the remote aspect of TD provides an opportunity to practice global health sustainably and allows for continuity of care. Moreover, it may have an educational value: with the patients’ consent, medical students and residents can also participate in TD consults, gaining exposure to sNTDs and global health without having to travel. Importantly, TD can also help general practitioners (GPs) recognize lesions associated with sNTDs endemic to the region where they practice. Such an application of TD was successfully applied in the United Kingdom [31].

The cost savings of TD should also be considered. Among the evaluated studies, 3 evaluated the financial aspect of TD implementation: Greisman et al reported TD in Guatemala and Uganda to be entirely feasible from a financial standpoint [17]; Cutler et al reported the cost of managing a TD platform in Haiti to be only US$5 per month [24]; and Messagier et al reported that TD mitigated healthcare expenses for more than 50% of patients in French Guiana [23]. As sNTDs affect the world’s most indigent, cost represents a significant barrier to care. TD may be a cost-effective way to expand care to individuals suffering from sNTDs.

Another advantage of TD is that it can be leveraged with mobile applications and artificial intelligence (AI). Although none of the studies we reviewed employed mobile applications and AI in conjunction with TD, we acknowledge the potential synergy of combining these technologies. Recently, Carrion et al reviewed the utility of mobile applications in mitigating the burden of sNTDs and although they concluded that numerous barriers to widespread mobile health implementation remain, their review demonstrates that creative mobile technologies, some with a modicum of success, do exist and can help curb the morbidity associated with sNTDs [32]. Adding AI can further potentiate the power of TD. A study conducted in the Philippines by Velasco et al showed that a neural network was able to diagnose common skin conditions with up to 94% accuracy [33]. Altogether, the combination of TD, mobile technologies, and AI represents a potent technological triad that may be used to control sNTDs.

The applications of TD in managing sNTDs that arise in non-community settings should also be acknowledged. While 19/20 studies we reviewed apply TD in the management of sNTDs in local community settings, the study by Hwang et al shows that TD can be effectively utilized to manage sNTDs that arise in military deployments [8]. TD also has the potential to be used in the management of sNTDs in refugee situations, and such an application of TD has been suggested in the treatment of skin disease in the refugee population in Europe [34] and the Rohingya in Bangladesh [35]. Furthermore, TD has the potential to diagnose non-endemic cases of sNTDs. In an increasingly interconnected world affected by rising global temperatures, the potential of sNTDs to present in regions outside the tropics has become a valid concern. In 2018, Hotez summarized how recent changes in climate, globalization, and urbanization have spurred the surge of NTDs in Texas [36]. Under these new circumstances, TD may acquire a truly global scope in the management of sNTDs; for example, in 2019 a woman who had recently immigrated to the United States from Brazil was diagnosed with leprosy using TD [37].

### Teledermatology and its Limitations

Inadequate image quality represents a major concern for TD. Six of the 20 studies we reviewed identified poor image quality as a barrier to diagnosis. Furthermore, TD works mostly as a triage system, and while it has the ability to identify patients that promptly need dermatologic attention, additional diagnostic procedures, such as biopsies, potassium hydroxide (KOH) preparations, dermoscopy, microscopy, and analysis under Wood’s lamp must all be sacrificed in an entirely TD model of care. In 10/20 studies, further diagnostic evaluation was recommended following a TD consult, and 2/20 studies indicated a lack of access to further diagnostic procedures. Many sNTDs may present ambiguously, necessitating further evaluation: microscopy for Buruli ulcer, cutaneous leishmaniasis, and lymphatic filariasis; biopsy for leprosy; and KOH preparations for fungal infections [38]. In developing countries where sNTDs are endemic and resources are scarce, further studies may not be possible. In such cases, a TD consult could potentially cause more harm than good by leaving the patient in a state of anxiety and uncertainty. Furthermore, only 4/20 studies mentioned any kind of patient follow-up; thus, the long-term efficacy of TD remains unknown.

Additionally, in 7/20 studies, the consulting dermatologist was located in a foreign location. As opposed to local dermatologists, dermatologists in more remote locations may not have a nuanced understanding of local disease epidemiology and available diagnostic techniques and treatments. This lack of knowledge could lead not just to potentially incorrect diagnoses but also recommendation of unavailable treatments.

The ethico-legal aspects of TD implementation must also be considered. Patient privacy represents a valid concern for TD implementation. Impressively, 14/20 studies acknowledged the issue of encryption and made an attempt at preserving patient confidentiality. The studies we reviewed utilized a myriad of platforms to perform TD consults, including Facebook [12], WhatsApp [26], Dropbox [20], and Tango [10], but the encryption underlying these platforms remains unclear. In fact, WhatsApp has been deemed inappropriate for telemedicine due to concerns about privacy breaches [39]. As TD use has increased during the COVID-19 pandemic, WhatsApp has come under further scrutiny. Brunasso and Massone point out that WhatsApp is not compliant with the European Union’s General Data Protection Regulation and that caution must be exercised in utilizing TD platforms [40]. Ensuring confidentiality is important, as individuals afflicted with sNTDs comprise a particularly vulnerable population and may be unable to advocate for themselves. Legal barriers to TD implementation also exist globally and are more pronounced in developing countries. Cutler et al addressed these limitations in their study on TD implementation in Haiti, citing that the legal framework surrounding telemedicine licensure and malpractice is nascent [24]. The control of sNTDs through TD must not come at the expense of ethical and legal transgressions.

Finally, cultural barriers to widespread TD utilization exist. From Saudi Arabia, Kaliyadan et al reported that 14% of patients refused to have their skin photographed, citing religious and social reasons [10]. While none of the other studies we reviewed evaluate the cultural barriers to TD implementation, it is possible that the hesitancy towards TD use exists in other countries afflicted by sNTDs, many of which embrace conservative cultures. Moreover, this cultural hesitancy towards TD acceptance may be amplified when the encryption of TD platforms is tenuous. Thus, the cultural milieu limiting TD use must not be overlooked.

### Teledermatology: One Piece of the Puzzle

The tremendous potential for TD is tempered by its limitations; alone, it is unlikely to eliminate sNTDs. Mass drug administration [41], advocacy, policy changes, involvement of key stakeholders, and greater investment in research have all been cited as elements essential to the control of sNTDs [42]. The community dermatology program in Guerrero, Mexico serves as an example of this integrated approach to managing sNTDs: TD is combined with education, mobilization of healthcare personnel, and involvement of local and international institutions to mitigate the burden of mycetoma [43]. Therefore, a more realistic picture of sNTD control is one where TD occupies one piece of the puzzle of sNTD eradication.

### Review Limitations

As we only considered published journal articles in our literature search, we recognize publication bias is a limitation to our review. Furthermore, only the sNTDs articulated by the WHO were used in our search criteria; however, other NTDs also present with cutaneous manifestations that can be evaluated by dermatologists. For instance, reactivated Chagas disease may present as cellulitic plaques, ulcers, necrotic eschars, and panniculitis [44]. Thus, our review may not exhaustively capture the application of TD in the management of all sNTDs.

## Conclusion

sNTDs represent a group of stigmatizing, poverty promoting diseases that can be effectively targeted by TD. TD can blunt the burden of sNTDs by expanding access to care in a manner that is both sustainable and cost-efficient. TD can also be deployed in combination with mobile health strategies and AI and used in non-community settings. However, poor image quality and the need for further diagnostic tests exemplify the limitations of the TD platform in the management of sNTDs. Ethico-legal and socio-cultural elements also constitute road-blocks to the global acceptance of TD. TD should feature as a major, but not the only, component in the strategy to eradicate sNTDs.

## Figures and Tables

**Table 1:** Literature Assessing TD Intervention in sNTD Control

Reference	Country/Region	sNTD	Location of Consulting Dermatologist	Study Size	Major Finding(s)
Baze, 2011 [11]	Honduras	FI, scabies	Foreign location	105	91% patient satisfaction; high dermatologist satisfaction; 4.3/5 image quality
Garcia-Romero et al, 2011[12]	Mexico	Scabies	Local jurisdiction	44	75% clinical improvement following TD consult
Tsang & Kovarik, 2011 [13]	Sub-Saharan Africa	Leprosy, LF, FI	Foreign location	55	58% correlation between TD and pathological analysis
Oseit-tutu et al, 2013 [14]	Ghana	FI	Local jurisdiction	34	79% concordance between FTF and TD consults
Montazeri et al, 2013 [15]	Iran	CL, FI	Local jurisdiction	91	85% concordance between FTF and TD consults
Smith et al, 2013 [16]	Kenya	FI	Local jurisdiction	32	Mean sensitivity of 73% and specificity of 83% for diagnosing tinea infections via TD
Kaliyadan et al, 2013 [10]	Saudi Arabia	FI	Local jurisdiction	166	95% concordance between FTF and TD consults
Greisman et al, 2014 [17]	Guatemala and Uganda	BU, CL, FI, LF, scabies	Foreign location	93	TD rectified GP diagnoses in 56% of cases
Hwang et al, 2014 [8]	U.S. Military Facilities	BU, CL, scabies, FI	Foreign location	658	98% of consults answered in 24 hours; 46 evacuations avoided and 41 evacuations facilitated due to TD consult
Lipoff et al, 2015 [18]	Sub-Saharan Africa	FI	Foreign location	1229	60% concordance between dermatologist and clinicians submitting images
Patro et al, 2015 [9]	India	FI, scabies	Local jurisdiction	206	56% concordance between FTF visit conducted by GP and TD
Nguygen et al, 2017 [19]	Cameroon	FI, leprosy, LF	Foreign location	145	Acceptable concordance between diagnosis as made by TD and light microscopy
Saleh et al, 2017 [20]	Egypt	FI	Local jurisdiction	600	87% concordance between FTF and TD consults
Ismail et al, 2018 [21]	Afghanistan	CL, FI, scabies	Local jurisdiction	326	Images of sufficient quality to render diagnoses in 94% of consults
Faye et al, 2018 [22]	Mali	FI, leprosy, scabies	Local jurisdiction	180	96% of patients properly managed via TD; mean time to dermatologist response was 32 hours
Messagier et al, 2019 [23]	French Guiana	CL, FI, leprosy, scabies	Local jurisdiction	254	85% satisfaction from users; 92% were able to be managed in peripheral health centers
Cutler et al, 2019 [24]	Haiti	FI, scabies	Foreign location	101	Average time from intake to case closure was 1.67 days; average diagnostic concordance between Haitian providers and U.S. dermatologists was 69%
Malmontent et al, 2020 [25]	French Guiana	Leprosy	Local jurisdiction	52*	TD used to solve four cases of leprosy
Singhal et al, 2020 [26]	India	FI; scabies	Local jurisdiction	520	9% of patients could not be assessed due to poor image quality; poor patient compliance to treatment following TD consult also noted
Lee et al, 2021 [27]	Taiwan	FI; scabies	Local jurisdiction	426	Subjective patient improvement >75% year-round and case closure rate >85% year-round

BU=Buruli ulcer; CL=cutaneous leishmaniasis; FI=fungal infections; FTF=face to face; GP=general practitioner; LF=lymphatic filariasis; sNTD=skin neglected tropical diseases; TD=teledermatology.

While the total number of cases for this study was 639, TD was used in only 52 cases.
